# Origin of Monocytes/Macrophages Contributing to Chronic Inflammation in Chagas Disease: SIRT1 Inhibition of FAK-NFκB-Dependent Proliferation and Proinflammatory Activation of Macrophages

**DOI:** 10.3390/cells9010080

**Published:** 2019-12-28

**Authors:** Xianxiu Wan, Imran Hussain Chowdhury, Zuliang Jie, Subhadip Choudhuri, Nisha Jain Garg

**Affiliations:** 1 Department of Microbiology and Immunology, University of Texas Medical Branch (UTMB), Galveston, TX 77550, USA; xianxiuwan@gmail.com (X.W.); ihchowdh@utmb.edu (I.H.C.); zjie@mdanderson.org (Z.J.); suchoudh@utmb.edu (S.C.); 2 Institute for Human Infections and Immunity, UTMB, Galveston, TX 77550, USA

**Keywords:** macrophage progenitors, ROS, *Trypanosoma cruzi*, SIRT1, Chagas disease, chronic inflammation

## Abstract

Background: *Trypanosoma cruzi (Tc)* causes Chagas disease (CD) that is the most frequent cause of heart failure in Latin America. TNF-α^+^ monocytes/macrophages (Mo/Mφ) are associated with inflammatory pathology in chronic CD. In this study, we determined the progenitor lineage of Mo/Mφ contributing to inflammation and examined the regulatory role of SIRT1 in modulating the Mo/Mφ response in Chagas disease. Methods and Results: C57BL/6 mice were infected with *Tc*, treated with SIRT1 agonist (SRT1720) after control of acute parasitemia, and monitored during chronic phase (150 days post-infection). Flow cytometry studies showed an increase in maturation of bone marrow hematopoietic stem cell (HSC)-derived Mo of proinflammatory and anti-inflammatory phenotype in acutely- and chronically-infected mice; however, these cells were not increased in splenic compartment of infected mice. Instead, yolk-sac-derived CD11b^+^ F4/80^+^ Mo/Mφ were increased in sinusoidal compartment of Chagas mice. The splenic CD11b^+^ F4/80^+^ Mo/Mφ of Chagas (vs. control) mice exhibited increased mRNA, protein, and surface expression of markers of proinflammatory phenotype (CD80^+^/CD64^+^ > CD200^+^/CD206^+^) associated with proinflammatory cytokines response (IL-6+TNF-α >> Arg-1+IL-10), and these were also detected in the myocardium of chronically infected mice. Infected mice treated with SRT1720 (vs. infected/untreated) exhibited decreased splenic expansion and myocardial infiltration of proinflammatory Mo/Mφ. SRT1720 did not alter the inherent capability of splenic Mo/Mφ of Chagas mice to respond to pathogen stimulus. Instead, SRT1720 dampened the *Tc*-induced increase in the expression and/or phosphorylation of focal adhesion kinase (FAK) and downstream transcription factors (Pu.1, c-Myb, and Runx1) involved in Mφ proliferation and migration and Notch1 involved in functional activation. Studies in cultured Mφ confirmed the agonistic effects of SIRT1 in controlling the *Tc*-induced, FAK-dependent increase in the expression of transcription factors and showed that SIRT1 agonist and FAK inhibitor abrogated the NF-κB transcriptional activity and inflammatory cytokine gene expression in *Tc*-infected Mφ. Conclusions: The proinflammatory Mo/Mφ of yolk sac origin drive the splenic and tissue inflammatory response in chronic CD. SRT1720 reprogrammed the *Tc*-induced FAK-dependent transcription factors involved in Mφ proliferation and proinflammatory activation in Chagas disease.

## 1. Introduction

*Trypanosoma cruzi* (*T. cruzi* or *Tc*) is the etiological agent of Chagas disease (CD) that is the most frequent cause of heart failure and sudden death in Latin America [1]. The parasite can infect a variety of host cells. Macrophages (Mφ) respond to invading pathogen by proinflammatory cytokines’ expression; and superoxide and nitric oxide (NO) production by the NADPH oxidase (NOX2) and inducible nitric oxide synthase (iNOS) enzymes, respectively [2,3]. The proinflammatory Mφ response is essential for intracellular parasite killing, yet it is not sufficient to prevent the spread of the parasite, and host often faces parasite persistence (reviewed in [4]). With progression to chronic disease phase, infected humans and animals are burdened by peripheral and myocardial inflammatory responses. The Th1, CD8^+^T cells, and TNF-α^+^ monocytes are often detected in peripheral blood of clinically symptomatic Chagas patients [5] and are believed to drive the cardiac pathology [6]. The factors that drive progenitor cells to support Mφ response against acute *T. cruzi* infection and during chronic disease phase are not described.

Monocytes (Mo), Mφ, and dendritic cells are the mononuclear phagocytic cells that together constitute monocyte phagocyte system (MPS) [7]. The hematopoietic stem cells (HSC) in fetal liver and adult bone marrow (BM) develop into monocytes and are released in blood. The BM- and blood- progenitor cells are directed towards mononuclear phagocyte lineage by colony stimulating factor and depending on the additional stimuli they are exposed to, differentiate into dendritic cells and/or mature Mo. Whether same population of BM progenitor cells differentiate into dendritic cells and Mo, and how this response changes in the presence of infectious agent (e.g., *T. cruzi*) is not defined. Splenic Mo/Mφ are generally derived from embryonic yolk sac and can be complemented by BM Mo. Whether BM Mo complement the prenatally established Mo/Mφ compartment in spleen and tissues in response to infection by *T. cruzi* is not studied.

The classical, proinflammatory activation of Mφ with LPS, IFN-γ, and GM-CSF is shown to be associated with enhanced cytotoxic and anti-tumor properties, and these were named as M1 Mφ (in alignment with Th1 T cells). The alternatively activated Mφ are recognized to cover a continuum of functional states and are sub-grouped as M2a (induced by IL-4 and IL-13), M2b (induced by immune complexes, TLRs and IL-1R ligands), and M2c (induced by IL-10, glucocorticoids) [2]. The M2 Mφ are involved in endocytic clearance of mannosylated ligands and reduction of proinflammatory cytokine secretion [8,9], and they play a central role in wound healing, fibrosis, tumor progression, and immune regulation [10].

Sirtuin 1 (SIRT1) is a highly conserved member of the family of NAD^+^-dependent Sir2 histone deacetylases. It utilizes NAD^+^ substrate and integrates mitochondrial metabolism and inflammation [11]. SRT1720 is a potent SIRT1 agonist that binds to the SIRT1 enzyme-peptide substrate complex, and it is shown to enhance deacetylation of SIRT1 target proteins in both cells and animal models [12,13]. We have recently found that treatment with SRT1720 suppressed the oxidative and inflammatory stress in chronically infected mice [14]. Whether beneficial effects of SRT1720 in modulating inflammatory pathology were delivered through silencing of the proinflammatory Mo/Mφ in CD is not known.

In this study, we aimed to determine the role of HSC and embryonic yolk sac progenitor cells in developing the proinflammatory Mo/Mφ response during *T. cruzi* infection and CD. We also evaluated if and how SIRT1 agonist might silence the proinflammatory polarization and proliferation of Mo/Mφ in CD. For this, we infected C57BL/6 mice with *T. cruzi*, and monitored the BM, splenic, and cardiac progenitor and mature Mo/Mφ profile during acute and chronic phases of infection and disease development. In some experiments, at the end of acute parasitemia, mice were given a short-term SRT1720 treatment (3-weeks total), and then monitored in chronic disease phase that succeeded at 6 months post-infection. We have used flow cytometry and biochemical and molecular approaches to study the source of proinflammatory Mo/Mφ in Chagas mice, and the SIRT1-dependent mechanisms in reprogramming the Mφ response in CD.

## 2. Material and Methods

### 2.1. Ethics Statement

All animal experiments were performed by following the NIH guidelines for Care and Use of Experimental Animals, and in accordance with protocols approved by the Institutional Animal Care and Use Committee at the University of Texas Medical Branch, Galveston (protocol number: 0805029, 05/05/2019). All experiments were conducted in ABSL2/BSL2-approved laboratory and all personnel have received appropriate ABSL2/BSL2 training.

### 2.2. Mice, Parasites, and Cell Culture

C57BL/6 mice (6 weeks old, females) were purchased from Jackson Laboratory (Bar Harbor, ME, USA). Trypomastigotes of *T. cruzi* (SylvioX10/4 strain, ATCC 50823, Manassas, VA, USA) were maintained and propagated by continuous in vitro passage in C2C12 cells. Mice were infected with *T. cruzi* (10,000 trypomastigotes/mouse, intraperitoneal). These mice exhibit acute parasitemia phase up to 40 days’ and chronic disease phase by 150 days’ post-infection (pi). Some mice were treated for short-term with SRT1720 (S1129, Selleck Chemicals, Houston, TX, USA, 1-mg per 100 µL per mouse, three times per week for three weeks). Specifically, SRT1720 treatment began after the control of acute parasitemia (i.e., during the 45–66 days’ pi period) and mice were euthanized at 150 days post-infection. Sera/plasma and tissue samples were stored at −80 °C. Bone marrow (BM) cells from femurs and splenic cells were isolated following a standard protocol [15], and either used immediately or stored in freezing media (90% FBS/10% DMSO) in liquid nitrogen.

RAW 264.7 Mφ (ATCC TIB-71) were propagated in high-glucose Dulbecco’s modified Eagle’s medium (DMEM) with glutamine containing 10% heat-inactivated fetal bovine serum (FBS) (Invitrogen, Carlsbad, CA, USA) and penicillin/streptomycin solution (100-μg each antibiotic per ml, Corning, NY, USA). For experimental use, RAW 264.7 Mφ were infected with *T. cruzi* trypomastigotes (cell-to-parasite ratio, 1:3) in presence or absence of SRT1720 (1 µM) or PF-562271 inhibitor of FAK (iFAK, 10 µM, Selleck Chemicals, Houston, TX, USA) for 4 h or 24 h.

Protein levels in all samples were determined by using the Bradford Protein Assay (Bio-Rad, Hercules CA, USA). All chemicals were of molecular grade (>99% pure) and purchased from Sigma-Aldrich (St. Louis, MO, USA).

### 2.3. Flow Cytometry

To analyze the hematopoietic stem cell (HSC) derived Mo during CD, BM cells and splenocytes were isolated from normal, and acutely and chronically infected mice. Single cell suspensions were incubated with red blood cell (RBC) lysis buffer (Sigma, St. Louis, MO, USA), washed with 1× PBS, and then suspended in brilliant stain buffer. Cells (5 × 10^5^ per 100 µL) were incubated with anti-mouse CD16/CD32 Fc block (BD Biosciences, San Jose, CA, USA) for 10 min at 4 °C, washed twice, and resuspended in 50 µL of flow cytometry staining buffer (00-4222-26, eBioscience, San Diego, CA, USA). Cells were then labeled with a cocktail of fluorochrome-conjugated antibodies for 30 min at 4 °C in dark. Cells were washed, fixed with cytofix solution for 20 min, washed, suspended in stain buffer, and visualized in duplicate on a LSRII Fortessa Cell Analyzer (BD Biosciences, San Jose, CA, USA), acquiring 30–50,000 events in a live cell gate. Data were further analyzed using FlowJo software (ver. 10.5.3, Tree-Star, San Carlo, CA, USA). The median fluorescence intensity (MFI) was derived from fluorescence histograms and was adjusted for background with isotype-matched control.

To analyze the changes in embryonic yolk-sac Mo/Mφ in CD, splenocytes from normal, *Tc*-infected, and *Tc*/SRT1720-treated mice were incubated with RBC lysis buffer (Sigma-Aldrich, St. Louis, MO, USA), washed twice with cold 1× PBS, and then suspended in PBS containing 0.5% BSA and 0.02% sodium azide (1 × 10^5^ cells/100 µL). Isolated splenocytes were washed with staining buffer, and incubated with Fixable Viability Dye (eFluor 506, 65-0866-14, Thermo Fisher Scientific, Waltham, MA, USA) according to the manufacturer’s instructions. Cells were incubated with anti-CD16/CD32 Fc block IgG for 15 min at 4 °C, washed, and labeled for 30 min at 4 °C in dark with fluorochrome-conjugated antibodies against markers of Mφ activation. When used for intracellular staining, cells were first fixed with 2% paraformaldehyde, re-suspended in permeabilization buffer (0.1% saponin/1% FBS in PBS), and then utilized for staining with antibodies against intracellular antigens. Cells were analyzed by flow cytometry, as above. All antibodies used in this study are listed in Table S1 (Supplementary Materials).

### 2.4. Purification and In Vitro Stimulation of Splenic Mo/Mφ

Single cell suspensions of splenocytes were made in 1× PBS containing 0.5% BSA and 2 mM EDTA. Splenocytes (1 × 10^8^) were subjected to positive selection of CD11b^+^ Mo/Mφ by using the MACS Microbeads Technology (130-049-601, Miltenyi Biotec, San Diego, CA, USA). Briefly, Mo/Mφ in the splenic cell populations were magnetically labeled with CD11b microbeads and cells were passed through MACS separation column while placed in the magnetic field. The columns were then washed three times with 5 mL MACS buffer to remove unbound cells. To detach CD11b^+^ cells from the column, cells were washed with 5 mL MACS buffer away from the magnetic field.

Isolated Mo/Mφ were seeded in 12-well cell culture plates (5 × 10^5^ cells/mL) in RPMI-5% FBS, and in vitro stimulated with *T. cruzi* trypomastigotes (cell: parasite ratio, 1:3) for 24 h at 37 °C, 5% CO_2_. Cells and supernatants were used for various experiments.

### 2.5. Gene Expression Analysis

Splenic Mo/Mφ from normal, *Tc*-infected, and *Tc*/SRT1720-treated mice were isolated as above, and in vitro incubated for 24 h in presence or absence of *T. cruzi* (5 × 10^5^ cells/mL, cell: parasite ratio, 1:3). RAW 264.7 Mφ (1 × 10^6^ cells/mL) were infected with *T. cruzi* (cell: parasite ratio, 1:3), and incubated for 24 h in presence or absence of 1 µM SRT1720 or 10 µM FAK inhibitor. Cells (1 × 10^6^ cells/mL) were suspended in Trizol reagent (Invitrogen, Carlsbad, CA, USA), and total RNA was extracted and precipitated by chloroform/isopropanol/ethanol method. Total RNA was treated with RNase-free DNase I (AM2222, Ambion, Austin, TX, USA) to remove the contaminating DNA, and analyzed for quality (OD_260/280_ ratio > 1.8) and quantity (OD_260_ of 1 = 40 µg/mL RNA) by using a NanoDrop ND-1000 spectrophotometer (Thermo Fisher Scientific, Wilmington, DE, USA). Purified RNA (1 μg) was reverse transcribed using the iScript^TM^ cDNA synthesis kit (1708841, Bio-Rad, Hercules, CA, USA) and diluted 5-fold with nuclease free ddH_2_O. A real time quantitative PCR was performed in a 20 µL reaction containing 1 µL cDNA, 10 µL SYBR green master mix (Bio-Rad), and 500 nM of each of the gene-specific oligonucleotides on an iCycler thermal cycler. The thermal cycling conditions were as follows: denature at 95 °C for 15-s and anneal at 60 °C for 30-s for 40 cycles. Specific product amplification was confirmed in the melt curve analysis from 63 °C to 95 °C. The PCR base line subtracted curve fit mode was applied for threshold cycle (Ct), and mRNA level was calculated by using iCycler iQ real-time detection system software (Bio-Rad, Hercules, CA, USA). The threshold cycle (C_t_) values of target mRNAs were normalized to mean Ct values of *GAPDH* housekeeping gene, and the relative mRNA level of each target gene was calculated by 2^−ΔCt^ method [16]. The oligonucleotides used in this study are listed in Table S2.

### 2.6. Western Blotting

Heart tissues (tissue: buffer ratio, 1:10 *w*/*v*) were homogenized in 1X RIPA buffer containing 1 mM PMSF (9806, Cell Signaling, Danvers, MA, USA). Splenocytes, purified splenic Mo/Mφ, or RAW 264. 7 Mφ (±*T. cruzi* and SRT1720 or FAK inhibitor, as above) were lysed in RIPA buffer (Millipore Sigma, St. Louis, MO, USA). Homogenates were centrifuged at 10,000× *g*, and supernatants were used as protein lysates. For the preparation of nuclear and cytosolic fractions, cells (5 × 10^6^/mL) were incubated on ice for 30 min in buffer A (10 mM HEPES, pH 7.9, 10 mM NaCl, 0.1 mM EDTA, 0.1 mM EGTA, 1 mM DTT, 1 mM PMSF) containing 0.625% NP-40 and 1% protease inhibitor cocktail. Cell lysates were centrifuged at 4 °C at 10,000× *g* for 1 min and supernatants stored as a cytosolic fraction at −80 °C. Pellets were washed with buffer A containing 1.7 M sucrose, re-suspended in buffer B (20 mM HEPES pH 7.9, 0.4 M NaCl, 1 mM EDTA, 1 mM EGTA, 1 mM DTT, and 1 mM PMSF), and centrifuged at 4 °C at 13,000× *g* for 5 min. The resultant supernatants were stored at −80 °C as nuclear extracts. Protein lysates (30 μg) were electrophoresed on a 4–15% Mini-Protein TGX gel using a Mini-PROTEAN electrophoresis chamber (Bio-Rad), and proteins were transferred to a PVDF membrane by using a Criterion Trans-Blot System (Bio-Rad). Membranes were blocked with 50 mM Tris, 150 mM NaCl (TBS) containing 5% non-fat dry milk (NFDM), washed three times for 10 min each with TBS-0.1% Tween 20 (TBST), and incubated overnight at 4 °C with antibodies against SIRT1, FAK, phosphor-FAK (P-FAK), CD11b, F4/80, CD80, CD206, PU.1, RUNX1, c-Myb or GAPDH (Table S1). All antibodies from Santa Cruz were used at 1:200 dilutions and all other antibodies were used at 1:1000 dilution in TBST-5% NFDM. Membranes were washed, incubated with HRP-conjugated secondary antibody (1: 10,000 dilution, Southern Biotech, Birmingham, AL, USA), and images were acquired by using an ImageQuant LAS4000 system (GE Healthcare, Pittsburgh, MA, USA). Densitometry analysis of protein bands of interest was performed by using a Fluorchem HD2 Imaging System (Alpha-Innotech, San Leandro, CA, USA), and normalized against GAPDH levels.

### 2.7. Cytokines and ROS Release

Splenic Mo/Mφ were in vitro incubated for 24 h in presence or absence of *T. cruzi*, SRT1720 and FAK inhibitor. Culture supernatants were analyzed by using optEIA™ ELISA kits (Pharmingen, San Diego, CA, USA) according to the manufacturer’s instructions to examine the cytokines (TNF-α, IL-6, and IL-10) release. Standard curves were prepared using the recombinant cytokines.

For the measurement of reactive oxygen species (ROS) release, culture supernatants (50 μL/well) were added in triplicate to 96-well black flat-bottomed plates. Samples were mixed with 50 μL of 100 μM 10-acetyl-3,7-dihydroxyphenoxazine (Amplex Red, Molecular Probes, Eugene, OR, USA) and 50 μL of 0.1 U/mL horseradish peroxidase. The H_2_O_2_-dependent oxidation of Amplex Red to fluorescent resorufin (Ex563_nm_/Em587_nm_) was recorded by using a SpectraMax M2 microplate reader (Molecular Devices, San Jose, CA, USA) [17].

### 2.8. Immunohistochemistry

To visualize in situ population of Mo/Mφ, paraffin-embedded 5 µm heart tissue sections were deparaffinized, kept in 10 mM sodium citrate buffer (pH 6.0), and subjected to microwave heating for 10 min. Slides were then washed twice in PBS, and sequentially incubated for 30 min each with 5% normal goat serum to reduce non-specific staining and 3% H_2_O_2_ to inactivate endogenous peroxidase. Tissue sections were incubated overnight at 4 °C with mouse monoclonal anti-Mac1 (1:50 dilution) antibody. After washing, slides were incubated at room temperature for 30 min each with biotinylated anti-mouse IgG (1:100 dilution) and streptavidin-conjugated alkaline phosphatase, and red color was developed with a Red AP Kit I (SK-5100, Vector Laboratories, Burlingame, CA, USA). Slides were then subjected to another round of microwave heating in order to prevent an antibody cross-reaction and also improve antibody access to nuclei. Following a second pre-incubation step as above, tissue-sections were labeled overnight at 4 °C with rabbit anti-iNOS (1:50 dilution) or rabbit anti-arginase 1 (1:50 dilution) antibodies. After washing, slides were labeled with biotinylated anti-rabbit antibody (1:100 dilution) and streptavidin-HRP conjugate (1:100 dilution) and brown color was developed with a DAB Peroxidase (HRP) Substrate Kit (SK-4100, Vector Labs, Burlingame, CA, USA). All slides were counter-stained with methyl green (stains nuclei). All of the immunostained sections were scored by the H-score method based on an assessment of the staining intensity together with the percentage of cells staining positively [18,19]. Briefly, 10 fields were randomly chosen at 20× magnification. The staining intensity in the cells was scored as 0, 1^+^, 2^+^, and 3^+^ corresponding to the negative, weak, moderate, and intense staining, respectively. The total number of cells and cells stained at different intensity were counted, and the percentages of cells at different intensity were calculated in each field. The H-score was determined following the formula: [1 × (gradation obtained from % of positive cells at 1^+^ intensity area + 2 × (gradation obtained from % of positive cells at 2^+^ intensity area) + 3 × (gradation obtained from % of positive cells at 3^+^ intensity area].

### 2.9. Transfection and NFκB Activity by Dual Luciferase Assay

Transfection and dual luciferase assay were conducted by using a Transfection Collection NFκB Transient Pack (79268, BPS Biosciences, San Diego, CA, USA). Briefly, RAW Mφ (30,000 cells/well/100 μL BPS medium) were seeded in 96-well, clear bottom, tissue culture plates. Cells were transfected with 1 μL of NFκB reporter (consists NFκB reporter vector + constitutively expressing Renilla luciferase vector) or negative control reporter (non-inducible luciferase vector + Renilla luciferase vector) diluted in 15 μL of Opti MEM I medium and mixed with 0.35 µL of Lipofectamine 2000. After incubation for 24 h at 37 °C/5% CO_2_, cells were replenished with fresh BPS medium, and incubated with *Tc* for 4 h and 24 h in the presence or absence of 1 µM SRT1720 and 10 µM FAK inhibitor (individually or in combination). Firefly and Renilla luciferase activities were evaluated by using Dual Luciferase (Firefly-Renilla) Assay System (BPS Biosciences, San Diego, CA, USA), and the release of luminescence was recorded by using a Glomax 96 microplate luminometer (Promega, Madison, WI, USA). The relative luminescence for NFκB reporter (firefly luciferase), normalized to Renilla luciferase (determines transfection efficiency), was calculated as a measure of changes in NF-kB activity.

### 2.10. Data Analysis

Mice were randomly assigned to diverse groups (*n* = 5 mice per group per experiment). All murine samples (e.g., BM and splenic cells in flow cytometry, purified Mφ in gene expression and western blotting, tissue sections in immunohistochemistry studies) were analyzed in duplicate or more. In vitro experiments were also conducted at least twice, and at least two biological replicates were analyzed in duplicate in each experiment. Data were analyzed by the student’s *t* test (comparison of two groups) and one-way analysis of variance (ANOVA) with Tukey’s post hoc test (comparison of more than two groups) by using GraphPad Prism software (version 7, La Jolla, CA, USA). Significance at *p* value of < 0.05, *p* < 0.01, and *p* < 0.001 is annotated by one, two, and three symbol characters, respectively (* normal vs. *Tc*-infected groups, and ^ comparison between infected and infected/treated groups).

## 3. Results

### 3.1. HSC Progenitor Monocyte’s Response during Chagas Disease

**Figure 1:**
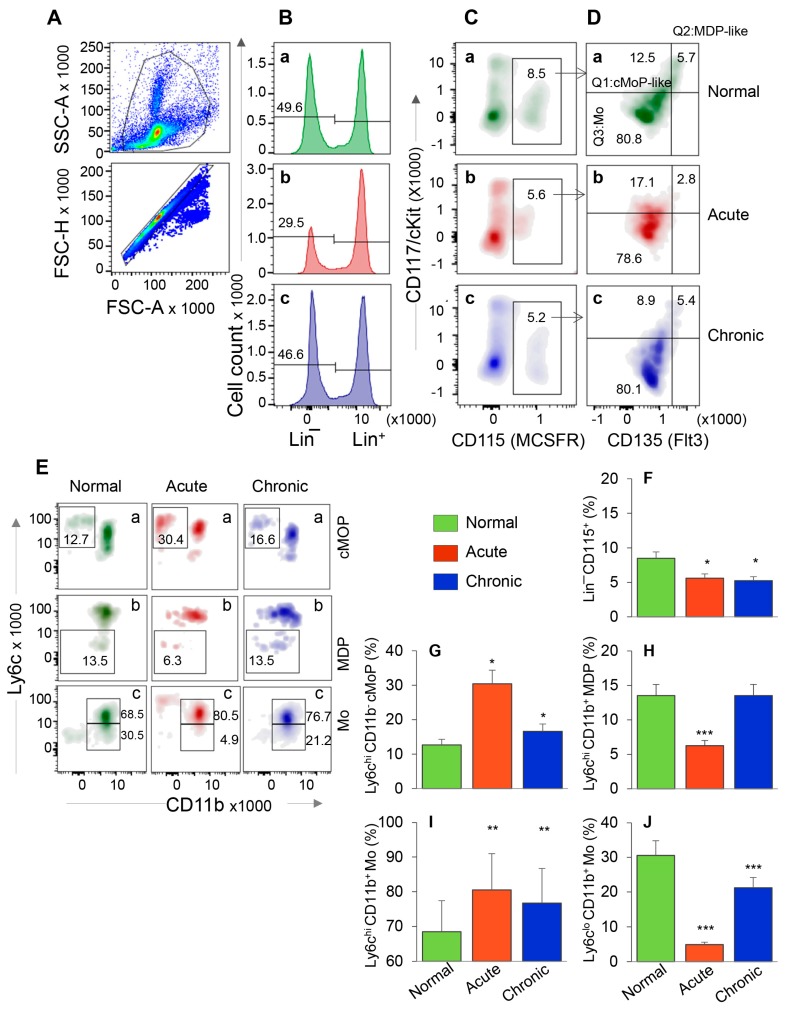
Bone marrow hematopoietic stem cell (HSC) monocyte progenitors’ response during *T. cruzi* infection and Chagas disease. C57BL/6 mice were infected with *T. cruzi* (10,000 trypomastigotes per mouse, i.p.) and euthanized at 30 days and 150 days post-infection (pi) corresponding to acute infection phase and chronic heart disease phase, respectively. Single cell suspensions of bone marrow cells were stained with fluorescence conjugated antibodies and analyzed by flow cytometry. After gating the cells of interest in forward and side scatter area (**A**, top panel), cells were further gated for forward scatter height to exclude doublets (**A**, bottom panel). From these, Lin^−^ HSCs (**B**) were gated for CD115 (MCSFR) in combination with CD117 (cKit) and CD135 (Flt3) (**C**,**D**). This gating resulted in identification of the frequencies of Lin^−^ CD115^+^ monocyte progenitors (MoP) that exhibited CD117^+^ CD135^+^ Mo/DC progenitor (MDP) like, CD117^+^ CD135^−^ common MoP (cMoP) like, and CD117^−^ CD135^−^ Mo phenotypes. Each cell population from panel D was gated for CD11b to confirm their lineage and determine the proinflammatory (Ly6c^hi^) and patrolling (Ly6c^lo^) phenotype (**E**). Bar graphs show the mean percentages of MoPs (**F**), cMOPs (**G**), MDPs (**H**), and monocytes that were of Ly6c^hi^ (**I**) and Ly6c^lo^ (**J**) phenotype in acute and chronic (vs. normal) mice. Data are representative of two independent experiments (*n* = 4 mice per group per experiment, 2–3 evaluations per sample) and plotted as mean value ± SEM. Significance is annotated as * *Tc*-infected vs. non-infected. * *p* value < 0.05; ** *p* value < 0.01; and *** *p* value < 0.001.

We first evaluated the HSC progenitors’ response in BM and spleen tissues of mice during acute *T. cruzi* infection and chronic disease development. After gating the cells of interest in forward and side scatter area (Figure 1A, top panel), cells were further gated for forward scatter height to exclude doublets (Figure 1A, bottom panel). From these, Lin^−^ HSCs (Figure 1B) were gated for CD115 (MCSFR^+^) in combination with CD117 (cKit) and CD135 (Flt3) to evaluate the frequency of Lin^−^ CD115^+^ monocyte progenitors (MoP) that exhibited CD117^+^ CD135^+^ Mo/DC progenitor like (MDP-like), CD117^+^ CD135^−^ common monocyte progenitor like (cMoP-like), and CD117^−^ CD135^−^ Mo phenotypes (Figure 1C,D). Each subset population from Figure 1D was further screened for Ly6c and CD11b to confirm the MDP (CD117^+^ CD135^+^ Ly6c^−^ CD11b^−^) and cMoP (CD117^+^ CD135^−^ Ly6c^+^ CD11b^−^) phenotypes. Both MDP and cMoP cells are capable of proliferation, MDP cells can be precursor of both cMoP and Mo, and cMoP cells are committed to primarily produce mature Mo/Mφ [20]. Finally, the CD117^−^ CD135^−^ CD11b^+^ cells with Ly6c^hi^ and Ly6c^lo^ phenotype were qualified as classical/inflammatory and patrolling mature Mo, respectively.

The representative flow cytometry determination of frequencies of MDP, cMoP, and Ly6c^hi^ and Ly6c^lo^ BM Mo are shown in Figure 1A–E, and bar graphs (Figure 1F–J) show the mean ± SEM values for various cell populations in normal, and acutely and chronically infected mice. In normal murine bone marrow, ~50% of cells were Lin^−^ HSCs, of which 8.5% were CD115^+^ MoPs (Figure 1B,C—a panels). In comparison, acutely and chronically infected mice exhibited ~34–40% decline in Lin^-^ HSC and MoP frequencies (Figure 1B,C—compare b and c with a, Figure 1F, all, * *p* < 0.05). Further analysis of MoP cells showed that frequencies of cMoP-like and cMoP cells were increased by 37% and 155%, respectively, and those of MDP-like and MDP cells were decreased by 50% in acutely infected (vs. control) mice (Figure 1D,E—compare b vs. a, Figure 1G,H, all, * *p* < 0.05). In chronically infected mice, percentages of cMoP cells were increased by 31% (* *p* < 0.05), while we observed no changes in MDP-like/MDP cell frequencies as compared to the normal controls (Figure 1D,E—compare c vs. a, Figure 1G,H). Up to 80% of the Lin^−^ CD115^+^ BM cells exhibited monocytic lineage (CD117^−^ CD135^−^) in all mice (Figure 1D) that was confirmed by CD11b^+^ phenotype (Figure 1E(c)). Of these, Ly6c^hi^ Mo (classical/inflammatory) were maximal (acute > chronic > control, Figure 1E(c),I, all, ** *p* < 0.01), and LyC^lo^ Mo (patrolling) were minimal (control > chronic > acute, Figure 1E(c),J, all, *** *p* < 0.001) in acutely infected mice.

When analyzing the splenic cells by a similar flow cytometry approach as above, we found that Lin^−^ HSC were present at a very low frequency in normal and infected mice (Figure S1A,B) in comparison to the Lin^−^ HSC population detected in BM of these mice (3.6–8.7% vs. 29.5–49.6%, respectively, compare Figure S1C and Figure 1B). Further, HSC-derived cMoP, MDP, and Mo constituted <1% of the splenic cell population, and, therefore, did not provide us an opportunity to reproducibly monitor functional profile of HSC monocytes in spleen with disease progression (data not shown).

Together, the results presented in Figure 1 and Figure S1 suggested that HSC-derived cMoP that are the committed progenitors for the generation of mature Mo and the maturation of Mo to inflammatory phenotype were increased in BM of acutely- and chronically-infected mice. The finding that the frequencies of HSC-derived Mo/Mφ were not significantly increased in the splenic compartment during *Tc* infection suggest that the HSC Mo are not the major source of sinusoidal Mφ response during *Tc* infection and disease development.

### 3.2. Embryonic Mo/Mφ Response during T. cruzi Infection in Presence or Absence of SRT1720

**Figure 2:**
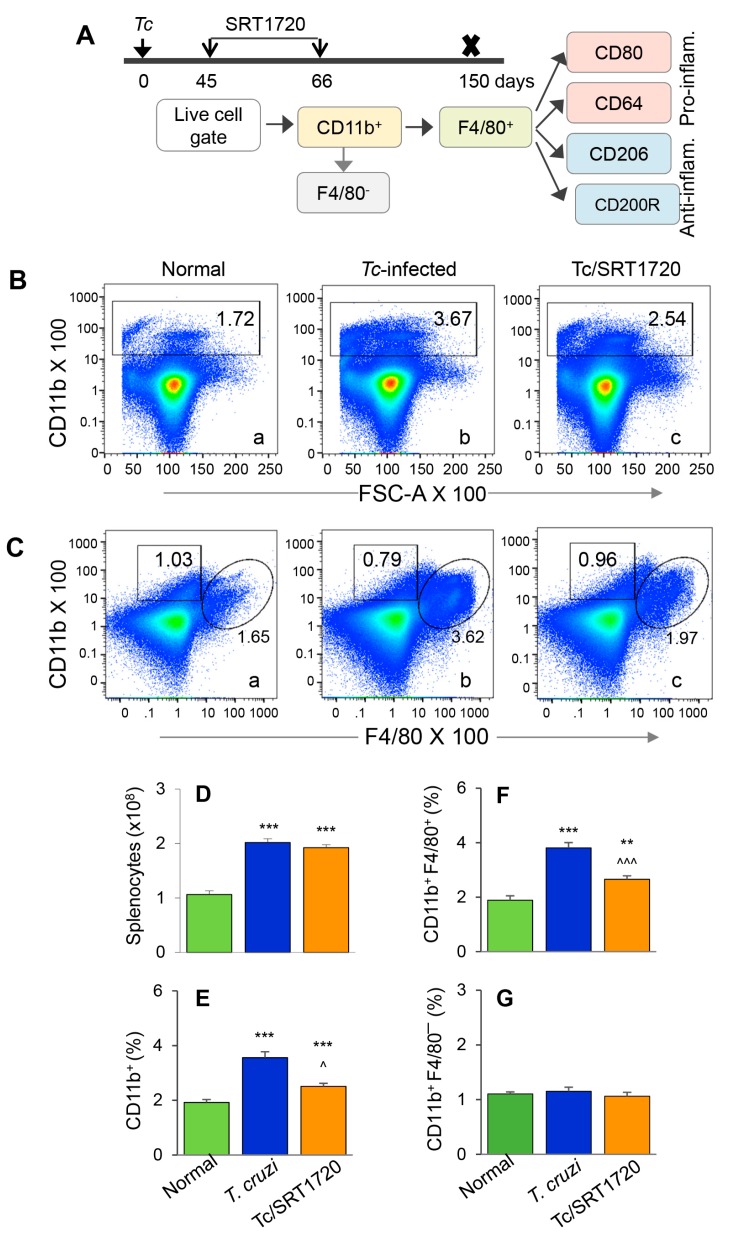
Splenic expansion of yolk sac Mo/Mφ in Chagas mice in presence or absence of SRT1720. (**A**) Diagram shows the scheme of experimental plan. Briefly, C57BL/6 mice were infected with *T. cruzi*. After control of acute parasitemic phase by immune system, mice were treated with SRT1720 (SIRT1 agonist) for three weeks, and then euthanized at 150 days post-infection. Single cell suspensions of splenic cells were stained with fluorescence-conjugated antibodies. Flow cytometry was performed to determine the frequencies of CD11b^+^ F4/80^−^ and CD11b^+^ F4/80^+^ macrophages. Each macrophage subset was then analyzed for markers of proinflammatory and anti-inflammatory phenotype. (**B**,**C**) Representative flow cytometry images of splenocytes from normal, infected, and infected/SRT1720-treated mice gated for CD11b in forward scatter (**B**) and CD11b/F4/80 together *(***C**) are shown. Bar graphs show the number of splenocytes (**D**), and mean percentages of CD11b^+^ (**E**), CD11b^+^ F4/80^+^ (**F**), and CD11b^+^ F4/80^−^ (**G**) splenocytes in normal, infected, and infected/SRT1720-treated mice. Data are representative of three independent experiments (*n* = 3–5 mice per group per experiment) and plotted as mean value ± SEM. Statistical significance is plotted as * non-infected vs. infected groups and ^ *Tc* only vs. *Tc*/SRT1720. ^ *p* value < 0.05; ** *p* value < 0.01; and *** or ^^^ *p* value < 0.001.

The F4/80 antigen is expressed by yolk sac (but not HSC) Mo/Mφ, and it was used as a marker to delineate the splenic population of Mo/Mφ in infected mice. Because the role of Mφ in chronic inflammatory pathology is not well delineated, we focused on examining the Mφ population in chronically infected mice. We also included SRT1720 treatment to examine the effect of SIRT1 on Mo/Mφ during chronic CD. The schematic for analyzing the splenic Mo/Mφ is presented in Figure 2A. Briefly, mice were infected, treated for short-term with SRT1720 (three times per week for three weeks), and euthanized in chronic phase at 150 days pi. Splenocytes were analyzed by flow cytometry for CD11b^+^ F4/80^+^ yolk sac macrophages that were further examined for markers of proinflammatory (M1) and anti-inflammatory (M2) phenotypes. Representative flow cytometry determination of frequencies of splenic CD11b^+^ F4/80^+^ Mφ in normal, infected, and infected/SRT1720-treated mice are presented in Figure 2B,C, and mean values from all analysis are plotted in bar graphs (Figure 2E–G). Our data showed the total splenic cell population in chronically infected mice was increased by 2-fold when compared to that noted in normal controls (Figure 2D, *** *p* < 0.001). The frequencies of CD11b^+^ Mo/Mφ and CD11b^+^ F4/80^+^ Mφ were increased by 85% and 102% in spleen of chronically infected (vs. control) mice (Figure 2B,C—compare a and b, Figure 2E,F, all, *** *p* < 0.001). Treatment with SRT1720 had no significant effect on splenic cell total population in Chagas mice (Figure 2D); however, it resulted in 28% and 30% decline in the percentages of CD11b^+^ and CD11b^+^ F4/80^+^ Mo/Mφ, respectively, in spleen of chronically infected mice (Figure 2B,C—compare b and c, Figure 2E,F, all, ^^ *p* < 0.01). These results suggested that a) embryonic cells are the major source of sinusoidal Mo/Mφ expansion during chronic *T. cruzi* infection, and b) SIRT1 agonist decreased the overall frequency of splenic F4/80^+^ Mφ in CD. Moreover, the observation of no changes in the percentages of CD11b^+^ F4/80^−^ cells (i.e., Mo of non-embryonic origin) in any of the treatment group (Figure 2C,G) provided further support to the findings in Figure 1, and confirmed that HSC-derived Mo did not contribute to sinusoidal Mφ response in Chagas disease.

**Figure 3:**
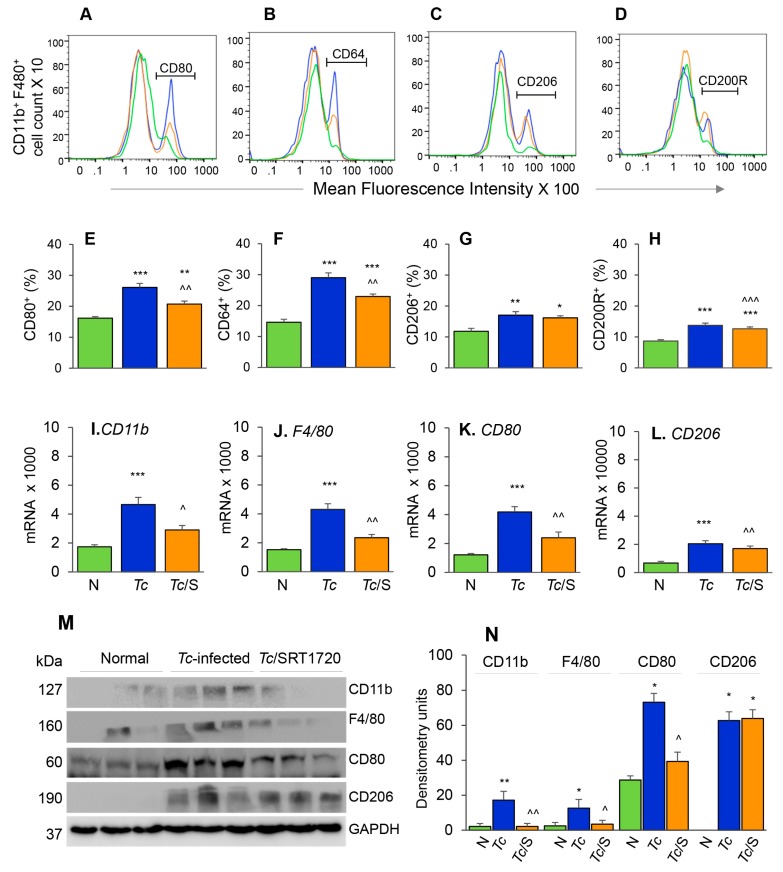
Phenotypic characterization of splenic Mφ in chronic Chagas mice in presence or absence of SRT1720. Mice were infected, SRT1720-treated, and euthanized as in Figure 2A. (**A**–**D**) Single cell suspension of spleen cells were stained with fluorescence-conjugated antibodies, gated for CD11b and F4/80 Mφ markers, and further examined for the expression of phenotypic markers of Mφ activation by flow cytometry. Shown are representative images of mean fluorescence intensity (*x*-axis) of splenocytes gated for the expression of CD80 (**A**), CD64 (**B**), CD206 (**C**), and CD200R (**D**) in normal (green), *Tc*-infected (blue), and infected/SRT1720-treated (orange) mice. (**E–H**) Flow cytometry was carried out in three independent experiments (*n* = 3–5 mice per group per experiment) and mean percentages of CD11b^+^ F4/80^+^ splenocytes that were CD80^+^ (**E**), CD64^+^ (**F**), CD206^+^ (**G**), and CD200R^+^ (**H**) in Chagas (±SRT1720) mice are plotted. (**I**–**L**) Real-time RT-qPCR analysis of mRNA levels for *CD11b, F4/80, CD80*, and *CD206* in the spleen of normal, *Tc*-infected, and infected/SRT1720-treated mice. For each target gene, Ct values were normalized to *GAPDH* expression. (**M**,**N**) Representative Western blot images for splenic levels of CD11b, F4/80, CD80, and CD206 in normal, infected, and infected/SRT1720-treated mice (loading control: GAPDH) are shown. Densitometry analyses of the Western blot bands for target proteins, normalized to GAPDH, are shown in panel ***N***. For panels I–N, data (mean value ± SEM) are representative of two independent experiments (*n* = 3–5 mice per group per experiment, duplicate observations per mouse). Statistical significance is plotted as * normal vs. infected and ^ *Tc* only vs. *Tc*/SRT1720. * or ^ *p* value < 0.05; ** or ^^ *p* value < 0.01; and *** or ^^^ *p* value < 0.001.

The phenotypic profile of the splenic Mo/Mφ in Chagas mice (±SRT1720) was evaluated by relative expression co-stimulatory molecule CD80 and the high-affinity Fc-γ receptor I (CD64) that are the robust phenotypic markers of M1 Mφ; and the CD200R and CD206 molecules that are associated with M2 Mφ [21]. As above, representative flow cytometry determination of frequencies of splenic Mφ that were CD80^+^, CD64^+^, CD206^+^ and CD200R^+^ in normal, infected, and infected/SRT1720-treated mice are presented in Figure 3A–D, and mean values are plotted in bar graphs (Figure 3E–H). These data showed 38–50% and 31–37% increase in the frequencies of the CD80^+^/CD64^+^ and CD206^+^/CD200R^+^ Mφ, respectively, within the CD11b^+^ F4/80^+^ splenic Mo/Mφ population in chronically infected (vs. control) mice (Figure 3E–H, all ** *p* < 0.01). SRT1720 treatment for 3-weeks led to 21% decline in *Tc*-induced increase in CD80^+^/CD64^+^ Mφ population in chronically infected mice (Figure 3E,F, all, ^^ *p* < 0.01), while no significant effects of SRT1720 were observed on splenic levels of CD206^+^/CD200R^+^ Mφ in CD mice (Figure 3G,H). The flow cytometry findings were consistent with RT-qPCR and Western blot analyses of the splenic levels of Mo/Mφ markers in CD (± SRT1720) mice. Splenic mRNA levels for *CD11b*, *F4/80*, *CD80*, and *CD206* were increased by 1.5–2.5-fold in infected (vs. control) mice (Figure 3I–L, all, *** *p* < 0.001), and SRT1720 treatment resulted in 38–45% decline in the mRNA levels of *CD11b*, *F4/80*, and *CD80* (Figure 3I–K all, ^ *p* < 0.05) and no change in *CD206* mRNA (Figure 3L) in splenocytes of Chagas mice. Likewise, protein levels for CD11b, F4/80, CD80, and CD206 were significantly increased in Chagas (vs. control) mice (Figure 3M,N, all, * *p* < 0.05), and SRT1720 treatment resulted in 47–88% decline in the protein levels of CD11b, F4/80, CD80 (Figure 3M,N, all, ^ *p* < 0.05) and no change in CD206 level in spleen of infected mice (Figure 3M,N). Together, the results presented in Figure 3, along with those presented in Figure 2, suggested that (a) the proliferation of embryonic yolk sac Mo/Mφ was associated with increased mRNA, protein, and surface expression of phenotypic markers of Mφ activation (M1 >> M2) in the spleen of chronically infected mice, and (b) SRT1720 decreased the frequency of Mo/Mφ of CD80^+^/CD64^+^ M1 phenotype in Chagas disease.

**Figure 4:**
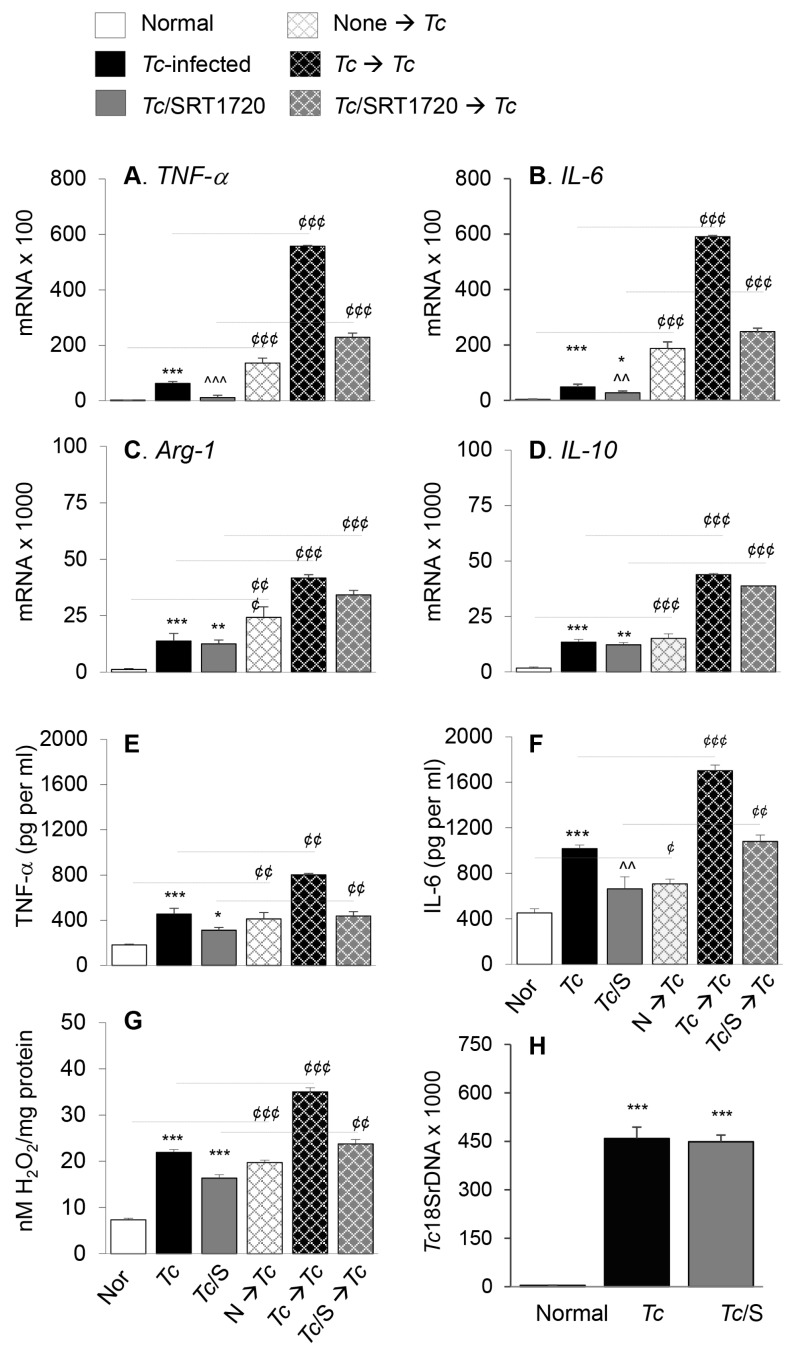
Functional activation of splenic Mo/Mφ in Chagas mice in presence or absence of SRT1720, and recall response to *T. cruzi*. Mice were infected with *T. cruzi* (*Tc*), treated with SRT1720 (S), and euthanized as in Figure 2A. Splenic Mo/Mφ were isolated by using MACS CD11b MicroBeads as described in Materials and Methods. Purified splenic Mo/Mφ from normal (none), *Tc*-infected and infected/SRT1720-treated mice were incubated with media alone (solid bars, no pattern) or with *Tc* (bars with cross-pattern) for 24 h to observe the recall response (**A**–**D**) Real time RT-qPCR evaluation of mRNA levels for *TNF-α* (**A**), *IL*-6 (**B**), *Arg-1* (**C**), and *IL-10* (**D**). For each target gene, Ct values were normalized to *GAPDH* expression. (**E**–**F**) An ELISA was performed to determine the TNF-α (**E**) and IL-6 (**F**) levels in cell-free supernatants. (**G**) ROS levels in the supernatants were measured by Amplex Red assay. For panels A–G, data (mean value ± SEM) are derived from two independent experiments (*n* = 3–5 mice per group per experiment, duplicate (**A**–**D**) or triplicate (**E**–**G**) observations per sample). (**H**) Level of parasite burden in splenic Mo/Mφ was determined by quantitative PCR amplification of the *Tc18SrDNA* sequence and normalized to *GAPDH* sequence. (*n* = 5 mice per group, duplicate observations per sample). Statistical significance is annotated as * normal vs. infected groups, ^ *Tc* vs. *Tc*/SRT1720, and ^¢^ splenic Mo/Mφ in vitro stimulated with *Tc* vs. matched non-stimulated Mo/Mφ. *^, ¢^ denote *p* value of < 0.05; **^,^ ^^^, ¢¢^ denote *p* value of < 0.01; and ***^,^ ^^^^, ¢¢¢^ denote *p* value of < 0.001.

Next, we determined if surface expression of physiological indicators of Mφ phenotype correlated with the proinflammatory versus anti-inflammatory functional profile of splenic Mφ in Chagas mice and whether SIRT1 agonist reprogrammed the intrinsic ability of Mφ to respond to pathogens. For this, we used anti-CD11b antibody-coated micro-beads to isolate the splenic Mo/Mφ from normal and chronically infected (±SRT1720) mice, and the isolated Mo/Mφ were either used directly or stimulated in presence or absence of *T. cruzi* for 24 h. RT-qPCR studies showed the mRNA levels of *TNF-α*, *IL-6*, *Arg-1*, and *IL-10* cytokines were increased by 27-fold, 13-fold, 10-fold, and 10-fold respectively, in splenic Mo/Mφ of Chagas (vs. control) mice (Figure 4A–D, all, *** *p* < 0.001). SRT1720 treatment provided 81% and 43% control of *TNF-α* and *IL-6* mRNA levels, respectively (Figure 4A,B, ^^^ *p* < 0.001), and had no significant effect on the *Arg-1* and *IL-10* mRNA levels (Figure 4C,D) in splenic Mo/Mφ of Chagas mice. Moreover, splenic Mo/Mφ of non-infected mice and infected (± SRT1720-treated) mice responded to in vitro stimulation with *Tc* antigen with 9–59-fold, 9–49-fold, 3–21-fold and 3–9-fold increase in mRNA levels of *TNF-α*, *IL-6*, *Arg-1*, and *IL-10*, respectively (vs. matched splenic Mo/Mφ without in vitro antigenic stimulation, Figure 4A–D, all, ^¢¢¢^ *p* < 0.001), and fold increase in cytokines mRNA level after in vitro stimulation with *Tc* was observed to be at a higher level in splenic Mo/Mφ of normal and *Tc*/SRT1720-treated mice.

We also evaluated the cytokines and H_2_O_2_ release as indicators of Mφ activation by an ELISA and Amplex Red assay, respectively. These results showed that splenic Mo/Mφ release of TNF-α, IL-6, and H_2_O_2_ were increased by 151%, 124%, and 199%, respectively, in Chagas (vs. normal control) mice (Figure 4E–G, all, *** *p* < 0.001), and controlled by 53%, 42%, and 76%, respectively, when infected mice were treated with SRT1720 (Figure 4E–G, all, ^^ *p* < 0.01). As above, splenic Mo/Mφ of all mice, irrespective of infection and SRT1720 treatment, were capable of responding to in vitro stimulation with *T. cruzi* by 140–230%, 100–240%, and 121–271% increase in TNF-α, IL-6, and H_2_O_2_ release, respectively (vs. matched splenic Mo/Mφ without in vitro antigenic stimulation, Figure 4E–G, all ^¢¢^ *p* < 0.01). No detectable level of IL-10 was observed in supernatants of any of the splenic Mo/Mφ before or after *Tc* antigenic stimulation. The persistence of parasite in splenic Mo/Mφ of Chagas mice was noted, and it was not changed by SRT1720 treatment (Figure 4H). Together, the results presented in Figure 4 suggested that a predominance of proinflammatory activation is displayed in splenic Mo/Mφ of chronically infected mice, and SRT1720 decreased the pro-inflammatory cytokine production. However, SRT1720 did not interfere with the ability of Mo/Mφ to respond to antigenic stimulus with proinflammatory cytokines and ROS production. Further, the findings of similar levels of parasite DNA in splenic Mo/Mφ of *Tc*-infected (±SRT1720) mice suggest that SRT1720 had no direct effect on parasite survival and persistence.

### 3.3. Macrophage Profile in the Myocardium of Chagas Mice in Presence or Absence of SRT1720

**Figure 5:**
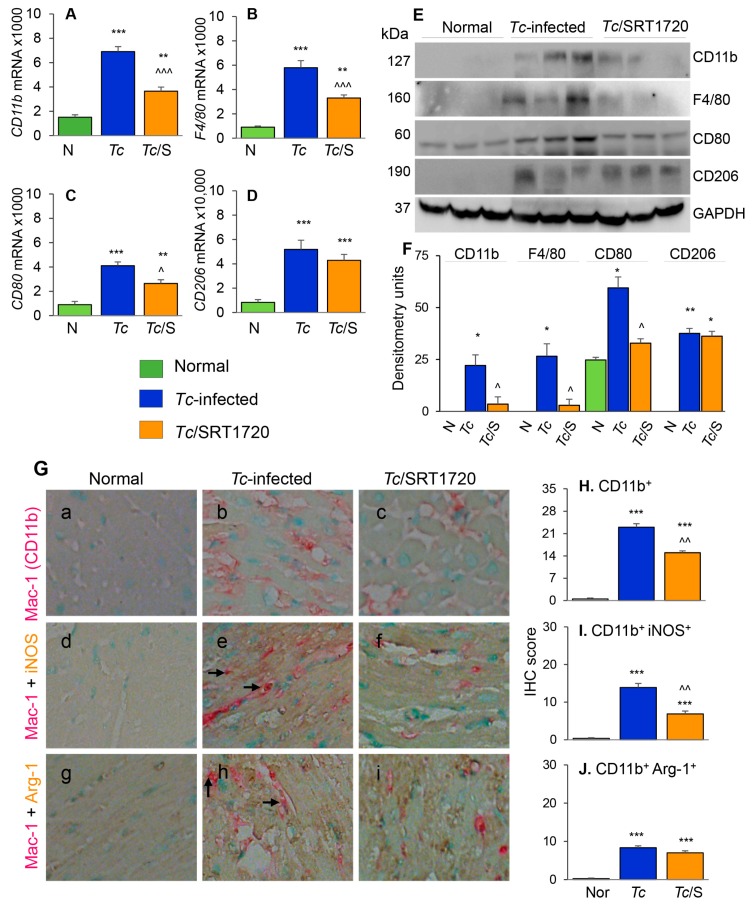
Phenotype of Mφ infiltrating the myocardium of Chagas mice treated (or not treated) with SRT1720. Mice were infected, SRT1720-treated, and euthanized as in Figure 2A. (**A**–**D**) Myocardial tissue levels of mRNAs for *CD11b, F4/80, CD80*, and *CD206* by real-time RT-qPCR. For each target gene, Ct values were normalized to *GAPDH* expression. (**E**,**F**) Shown are representative western blot images for cardiac levels of CD11b, F4/80, CD80, and CD206 in normal, Chagas, and infected/SRT1720-treated mice (loading control: GAPDH). Presence of dual bands for CD80 are likely due to post-translational modifications of the protein. Densitometry analyses of the western blot bands for target proteins, normalized to GAPDH, are shown in panel F. Data are presented as mean value ±SEM, derived from two independent experiments (*n* = 3–5 mice per group per experiment, at least duplicate observations per mouse). (**G**) Paraffin-embedded heart tissue sections were subjected to immuno-staining with antibodies against Mac1/CD11b (Mo/Mφ marker, pink color, (**G** (a–i)), iNOS (M1 Mo/Mφ marker, brown color, (**G** (d–f)), and Arg-1 (M2 Mo/Mφ marker, brown color, (**G** (g–i)). Shown are representative images of heart tissue of normal (**G** (a,d,g)), Chagas (**G** (b,e,h)), and infected/SRT1720-treated (**G**. (c,f,i)) mice. Arrows indicate the pink/brown staining of Mac1/CD11b with iNOS or Arg-1. (**H**–**J**) Bar graphs show average immunohistochemistry (IHC) score ± SEM for CD11b^+^ (**H**), CD11b^+^ iNOS^+^ (**I**), and CD11b^+^ Arg-1^+^ (**J**) phenotype in heart tissue sections (*n* = 3 mice per group, 2–3 tissue sections per mouse, 9–10 microscopic fields per tissue section). Statistical significance is plotted as * normal vs. infected groups, and ^ *Tc* vs. *Tc*/SRT1720. * or ^ *p* value < 0.05; ** or ^^ *p* value < 0.01; and *** or ^^^ *p* value < 0.001.

To further understand the effects of SIRT1 agonist in CD, we examined the tissue infiltration of Mo/Mφ in *T. cruzi*-infected (±SRT1720) mice by various approaches. The myocardial mRNA levels of CD11b, F4/80, CD80, and CD206 were increased by 3.5–5.3-fold in chronically infected (vs. control) mice (Figure 5A–D, all, *** *p* < 0.001). Likewise, myocardial CD11b, F4/80, CD80, and CD206 proteins were significantly increased in Chagas (vs. normal control) mice (Figure 5E,F, all, * *p* < 0.05). Treatment with SRT1720 resulted in 47–60% control of mRNA levels and >75% control of protein levels of CD11b, F4/80, and CD80 markers of Mo/Mφ in Chagas myocardium (Figure 5A–F, all, ^^^ *p* < 0.05), while no significant effects of SRT1720 were observed on myocardial levels of CD206 in Chagas mice (Figure 5D–F). Immunohistochemistry studies with dual staining for Mac1/CD11b (Mo/Mφ marker, red color) and iNOS (M1 Mo/Mφ marker, brown color) or Arg-1 (M2 Mo/Mφ marker) were also conducted to evaluate the changes in the myocardial Mo/Mφ population in Chagas mice. These data showed that myocardial CD11b^+^, CD11b^+^ iNOS^+^, and CD11b^+^ Arg-1^+^ cell populations were almost undetectable in normal control mice and increased by >20-fold in Chagas myocardium (Figure 5G—compare a, d, e with b, e, h, respectively, Figure 5H–J, all, *** *p* < 0.001). SRT1720 treatment resulted in 40% and 50% decline in myocardial infiltration of CD11b^+^ and CD11b^+^ iNOS^+^, Mo/Mφ in Chagas mice (Figure 5G—compare b and c and e and f, Figure 5H,I, all, ^^ *p* < 0.01). SRT1720 treatment did not alter the myocardial population of CD11b^+^Arg-1^+^ Mo/Mφ (Figure 5G—compare h and i, Figure 5J). In chronically infected mice, all of the iNOS^+^ and Arg-1^+^ cells appeared to be not overlapping with Mac1/CD11b^+^ Mo/Mφ, thus, indicating that iNOS and arginase-1 are also produced by other cell types in Chagas myocardium. Together, the results presented in Figure 5 suggested that yolk sac derived Mφ (Mac1/CD11b^+^ and F4/80^+^) of proinflammatory (CD80^+^, iNOS^+^) and immunomodulatory (CD206^+^ Arg-1^+^) phenotypes were significantly increased in the myocardium of infected mice, and SRT1720 shifted the immune balance towards anti-inflammatory/immunomodulatory Mo/Mφ response in Chagas disease.

### 3.4. SIRT1 Regulates FAK-Mediated Activation of Transcription Factors Involved in Mφ Response to Tc Infection

**Figure 6:**
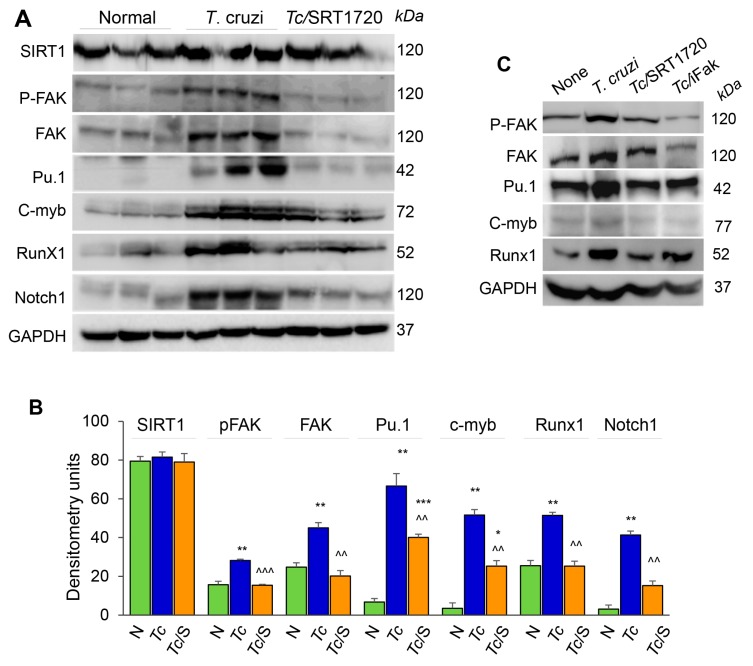
FAK phosphorylation and expression of transcriptional regulators of Mφ response during *Tc* infection in the presence or absence of SRT1720. (**A**,**B**) C57BL/6 mice were infected with *T. cruzi* (*Tc*), treated with SRT1720 (S), and euthanized as in Figure 2A, and splenic Mo/Mφ were purified as in Figure 4. (A) Shown are representative western blot images for splenic Mo/Mφ levels of SIRT1, phosphorylated FAK (P-FAK), total FAK, Pu.1, c-myb, Runx1 and Notch1 in normal, Chagas, and infected/SRT1720-treated mice (loading control: GAPDH). Presence of dual bands for Pu.1 are likely due to post-translational modifications of the protein. Densitometry analyses of the western blot bands for target proteins, normalized to GAPDH, are presented as mean value ± SEM in panel B and derived from three independent experiments (*n* > 3 mice per group per experiment). Statistical significance is plotted as * non-infected (N) vs. infected groups and ^ *Tc* vs. *Tc*/SRT1720. * *p* value < 0.05; ** or ^^ *p* value < 0.01; and *** or ^^^ *p* value < 0.001. (**C**) RAW 264.7 Mφ were incubated for 24 h with *T. cruzi* trypomastigotes (cell parasite ratio, 1:3) without or with SRT1720 (1 μM) or inhibitor of FAK (iFAK, 10 μM). Representative western blot images of P-FAK, total FAK, Pu.1, c-myb and Runx1 protein levels in non-infected, *Tc*-infected, infected/SRT1720-treated and infected/FAK inhibitor treated Mφ are shown (GAPDH, loading control).

Focal adhesion kinase (FAK), an intracellular tyrosine kinase, is implicated in regulating diverse cellular functions including cell adhesion, migration, invasion, polarity, proliferation, and survival [22,23]. Further, transcription factors, including Pu.1, c-myb, Runx1, and Notch1 (individually or in combination) are implicated in proliferation, tissue migration, and polarization of Mo/Mφ [24]. We, therefore, hypothesized that SIRT1 regulates Mφ response by targeting the FAK activation and downstream transcription factors. Western blotting showed that SIRT1 level was not changed in isolated splenic Mo/Mφ of chronically infected and infected/SRT1720-treated mice as compared to that noted in normal controls (Figure 6A,B). However, protein levels of FAK (total and phosphorylated), Pu.1, c-myb, Runx1, and Notch1 were increased by ~2-fold, 6-fold, 17-fold, 2-fold, and 10-fold, respectively, in the splenic Mo/Mφ of chronically infected (vs. normal) mice (Figure 6A,B, all, ** *p* < 0.01). Subsequently, SRT1720 treatment abolished the *Tc*-induced increase in FAK, P-FAK, and Runx1 levels, and resulted in >50% decline in the Pu.1, c-myb, and Notch1 levels in splenic Mo/Mφ of Chagas mice (Figure 6A,B, all, ^^ *p* < 0.01).

We confirmed the role of SIRT1 and FAK in signaling the expression of Mφ transcription factors by using in vitro cultured Mφ. RAW 264.7 Mφ were infected with *T. cruzi* trypomastigotes, and incubated in the presence or absence of SRT1720 or selective inhibitor of FAK (iFAK) for 24 h. Western blotting showed a significant increase in the expression and phosphorylation of FAK, and a potent increase in the expression of Pu.1 and Runx1 transcription factors in RAW Mφ infected with *Tc* (vs. non-infected controls, Figure 6C). The *Tc*-induced increase in FAK and P-FAK levels were decreased by 50% and 90% by SRT1720 and iFAK, respectively (Figure 6C). Both SRT1720 and iFAK also neutralized the *Tc*-induced increase in the expression of Pu.1, Runx1, and Notch1 (Figure 6C and data not shown). Together, these results suggested that SIRT1-FAK regulate the downstream transcription factors involved in Mφ maturation, survival, and activation during Chagas disease.

**Figure 7:**
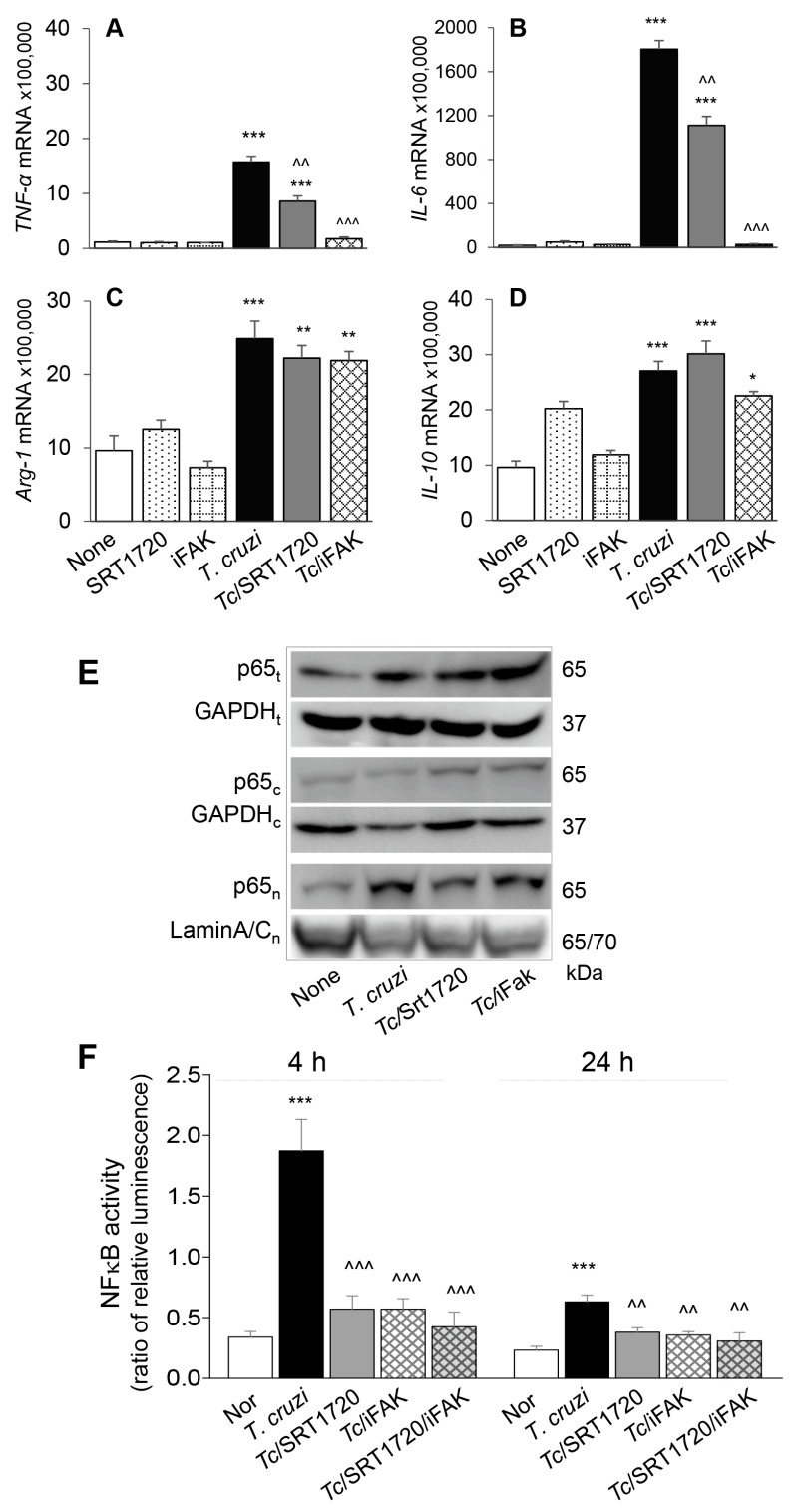
SIRT1-FAK (Sirtuin 1-focal adhesion kinase) regulate nuclear translocation of P65, NF-κB activity, and cytokines’ gene expression in Mφ infected by *T. cruzi*. (**A**–**D**) RAW 264.7 Mφ were incubated for 24 h with *T. cruzi* trypomastigotes (cell parasite ratio, 1: 3) in absence or presence of SRT1720 (1 μM) or inhibitor of FAK (iFAK, 10 μM). Gene expression analysis of *TNF-α (A), IL-6* (**B**), *IL-10* (**C**) and *Arg-1* (**D**) was performed by real-time RT-qPCR, and Ct values for target genes were normalized to *GAPDH* expression (three biological replicates per group, duplicate observations per sample). (**E**) RAW 264.7 Mφ were infected and treated with SRT1720 or FAK inhibitor, as above. Shown are representative immunoblots for NFκB p65 subunit in total (t) cell homogenates, and cytosolic (c) and nuclear (n) fractions of normal, infected, and infected/treated cells. GAPDH (cytosolic and total homogenates) and Lamin A/C (nuclear fractions) were analyzed as loading controls. (**F**) RAW 264.7 Mφ were transfected with NF-κB luciferase reporter and control renilla luciferase. Transfected cells were incubated with *Tc* (±SRT1720, FAK inhibitor) for 4 h and 24 h. Cells were then used in a Dual Luciferase (Firefly-Renilla) Assay, and the ratio of relative luminescence for firefly to renilla luciferase was calculated as a measure of NF-κB activity. In all experiments, non-infected cells treated similarly with SRT1720 or iFAK were used as controls. Data in bar graphs are representative of two independent experiments (three biological replicates per treatment) and presented as mean ± SEM. Statistical significance in all bar graphs is plotted as * non-infected (N) vs. infected groups, ^ *Tc* vs. *Tc* and SRT1720 and/or FAK inhibitor treatment. * *p* value < 0.05; ** or ^^ *p* value < 0.01; and *** or ^^^ *p* value < 0.001.

To confirm that SIRT1/FAK influence the functional activation of Mφ in response to *Tc* infection, we incubated RAW Mφ for 24 h with or without *Tc* (±SRT1720 or iFAK) and monitored the cytokines’ gene expression by RT-qPCR. These data showed 20-37-fold increase in *TNF-α* and *IL-6* and up to 2-fold increase in *Arg-1* and *IL-10* mRNA levels in Mφ infected by *Tc* (vs. controls, Figure 7A–D, all, *** *p* < 0.001). SRT1720 treatment resulted in ~45% decline in *Tc*-induced *TNF-α* and *IL-6* expression (Figure 7A,B, all, ^^ *p* < 0.01) while FAK inhibitor resulted in >85% control of *Tc*-induced increase in *TNF-α* and *IL-6* mRNA levels (Figure 7A,B, all, ^^^ *p* < = 0.001) in infected Mφ. SRT1720 and FAK inhibitor had no effect on cytokines’ gene expression in non-infected cells and did not alter the *Arg-1* and *IL-10* expression in *Tc*-infected cells (Figure 7C,D).

NFκB transcriptional factor is a critical regulator of Mφ proinflammatory activation. Rel A (p65) is an important subunit of activated NFκB dimers (p50/p65, p65/p65, and p65/c-Rel), and its nuclear translocation initiates the assembly and activation of NFκB transcriptional complex for proinflammatory gene expression. We, therefore, examined the nuclear translocation of p65 in RAW Mφ after 24 h incubation with *Tc* (±SRT1720 or iFAK). Western blotting showed that expression and nuclear translocation of p65 were increased by >3-fold in *Tc*-infected (vs. non-infected) RAW Mφ (Figure 7E). SRT1720 or iFAK had no effect on the extent of *Tc*-induced increase in p65 protein level; however, treatment with these molecules resulted in increased retention of p65 in the cytosol, and a corresponding decline in the nuclear translocation of p65 in infected Mφ (Figure 7E), thus, suggesting that SIRT1 and FAK exert their effect on proinflammatory Mφ activation through regulating the nuclear translocation of p65/RelA.

Finally, we performed a dual luciferase reporter assay to confirm the effects of SIRT1/FAK on NF-κB activity. Mφ incubated with *Tc* for 4 h exhibited 466% increase in NF-κB-luciferase activity (normalized to *Renilla* luciferase, *** *p* < 0.001) that was inhibited by >70% in presence of 1 μM SRT1720 and 10 μM FAK inhibitor (individually or in combination) (Figure 7F, all, ^^^ *p* < 0.001). At 24 h post-incubation, Mφ continued to respond to *Tc* with 174% increase in NF-κB-luciferase activity (*** *p* < 0.001) that was inhibited by 41–52% in presence of SRT1720 and FAK inhibitor (Figure 7F, all, ^^ *p* < 0.01). Together, the results presented in Figure 7, along with those presented in Figure 6, suggested that a decline in SIRT1 activity contributes to FAK-dependent increase in the expression of transcription factors required for proliferation and maturation of Mφ and increase in the activity of NFκB required for proinflammatory activation of Mφ in CD. Treatment with SIRT1 agonist suppressed the FAK phosphorylation, and FAK-dependent increase in Pu.1 and Runx1 involved in Mφ proliferation and polarization, and also suppressed the NFκB activity required for proinflammatory activation of Mφ in response to *Tc* infection. We surmise that SIRT1/FAK homeostasis offers a potential therapy for controlling the chronic inflammation in CD.

## 4. Discussion

During the early phase of infection, *T. cruzi* induces a broad inflammatory response that persists and contributes to the pathogenesis of CD [25]. Macrophages serve as the first responders to *T. cruzi* infection and play a significant role in the direct killing of *T. cruzi* [26]. Published reports [27,28] and our results in this study show that splenic and peritoneal Mc release high levels of TNF-α, IL-6, IL-1β, and some nitric oxide (NO) in response to acute *T. cruzi* infection. Acute parasitemia is controlled by innate and adaptive immune responses against *T. cruzi*; however, chronic inflammation persists in the host. Several studies have shown a systemic increase in TNF-α^+^ monocytes [5,29,30], oxidative stress (e.g., lipid hydroperoxides) [31,32], and an abundance of CD8^+^T cells that express inflammatory cytokines and cytotoxic molecules in Chagas patients with clinically symptomatic heart disease. Microscopic examination of tissues also routinely shows exaggerated inflammation in the heart that contributes to thrombosis, right bundle branch block, left ventricular dilation, and heart failure. In this study, we focused on evaluating if hematopoiesis in BM (vs. spleen) contributes to immune cells’ supply at sites of inflammation in Chagas disease. We also determined the molecular mechanism(s) that can be targeted to silence the pathological/proinflammatory Mφ response in chronic CD.

In mice, hematopoietic stem cells (HSCs) in fetal liver and adult BM differentiate to hematopoietic progenitors that produce Ly6c^hi^ Mo. Our flow cytometry results showed that acute and chronic infection by *T. cruzi* enhanced the BM frequencies of HSC-derived cMoP that are the committed progenitors for the generation of Mo/Mφ (Figure 1). The highest frequencies of CD11b^+^Ly6c^hi^ Mo in acutely infected mice was in alignment with their function in removal of damaged tissue and pathogens while the higher frequencies of CD11b^+^Ly6c^lo^ Mo noted in chronically infected mice was in alignment with their role in promoting tissue healing. Recent studies have also identified distinct splenic population of Ly6c^hi^CD11b^+^ Mo that coexist with, but is different from, Mφ [33]. These Ly6c^hi^CD11b^+^ Mo were shown to mobilize *en masse* after myocardial infarction in mice [33]. We did not detect a major change in the frequencies of Ly6c^hi^CD11b^+^ F4/80^-^ Mo/Mφ in the splenic compartment during *Tc* infection (Figure S1 and Figure 2D). Instead CD11b^+^ F4/80^+^ Mφ (yolk sac origin) were significantly increased in spleen and heart tissue of Chagas mice (Figure 2 and Figure 5). We, therefore, concluded that HSC derived Mo/Mφ are not the major source of increased population of sinusoidal and tissue resident Mφ during *T. cruzi* infection and CD. Further studies will be required to delineate if the HSC-derived Mo underwent apoptotic death or migrated to tissues other than spleen in Chagas disease.

Sirtuin 1 (SIRT1, chromatin remodeling response enzyme) and poly-(ADP) polymerase 1 (PARP-1, DNA damage response enzyme) are the most enzymatically active members of their respective families [34]. SIRT1 and PARP1 affect two key post-translational modifications: acetylation and ADP-ribosylation, respectively. These enzymes are transcriptionally and functionally connected due to their use of NAD^+^ substrate [35], SIRT1′s ability to deacetylate PARP1 and regulate PARP1 promoter [36], and PARP’s ability to influence SIRT1 promoter [37]. We have found that SIRT1 activity was decreased and PARP1 activity was increased in CD and inhibition of PARP1 or treatment with SRT1720 to enhance SIRT1 activity improved the left ventricular function in Chagas mice [14,38]. Further studies will be required to delineate the complex interconnectivity between these two proteins in CD. Yet, in the context of *T. cruzi* infection, we have found no direct effects of SIRT1 agonist and PARP1 inhibitor on parasite persistence (Figure 4H and [38]). Instead, it appears that PARP1 and SIRT1 balance the Mo/Mφ role for removal of cellular debris produced by *Tc* infection and prevent further tissue damage while promoting healing. Transcription factors, including Pu.1 (regulates proliferation, differentiation, or activation), c-myb (involved in differentiation and maturation of yolk-sac Mφ), and Runx1 (regulates growth and survival of Mφ) are involved in proliferation and tissue migration of Mo/Mφ, and all of these were up regulated and associated with increase in Mo/Mφ population in Chagas mice. Importantly, treatment with SRT1720 led to a significant control of *Tc*-induced increase in Pu.1, c-myb, Runx1 expression in spleen (Figure 6), and a > 50% decline in the frequencies of Mo/Mφ in spleen (Figure 2E) and heart (Figure 5G) of Chagas mice. These findings indicate that at least one mechanism by which SRT1720 may offer benefits in improving the heart function in Chagas disease is through inhibiting Mφ proliferation and migration and establishing the Mφ homeostasis in tissues. Indeed, others have shown the beneficial effects of resveratrol (SIRT1 agonist) in decreasing the Mφ migration to pancreas in a mouse model of diabetes [39] and mesenteric lymph nodes and lamina propria in a mouse model of colitis [40], while genetic deletion of SIRT1 resulted in increased Mφ infiltration in the synovial tissues in inflammatory arthritis [41]. Some studies also indicated that metabolites of NAD (generated by SIRT1 and PARP1 activity) interfere with differentiation of THP-1 cells along the monocytic path [42]; however, to the best of our knowledge, this is the first observation of SIRT1 regulation of Mφ proliferation in health and disease. How SIRT1 regulates Pu.1 (or other transcription factors) and the fate of Mφ in CD remains to be determined in future studies.

In addition to Mo/Mφ proliferation/migration, treatment with SRT1720 decreased the proinflammatory phenotypic (Figure 3) and functional (Figure 4 and Figure 5) activation of Mo/Mφ in spleen and heart tissue of Chagas mice. Though a large body of literature suggest that SIRT1 inhibits M1 Mφ polarization [43,44,45,46], it is not entirely clear as to how SIRT1 regulates Mo/Mφ activation. Our results indicate that SIRT1 did not change the inherent capacity of the Mφ to produce inflammatory cytokines in response to exposure to pathogen (Figure 4). Instead, SRT1720 decreased the expression of Notch1 (Figure 6) that can regulate Mφ response at multiple levels. Notch1 was reported to enhance IL-6, MCP-1, and TNF-α production in Mφ [47] and upregulate the expression of NF-κB subunits in BM hematopoietic progenitor cells [48]. SIRT1-depedent deacetylation of Notch1 diminished the LPS-mediated Mφ activation [49]. Thus, we surmise that SRT1720 treatment, at least partially, regulated the proinflammatory Mφ activation through inhibiting the Notch1 signaling pathway.

Focal adhesion kinase (FAK) is a 125 kDa non-receptor tyrosine kinase that is shown to be overexpressed in tumors, and play a significant role in adhesion, survival, motility, metastasis, angiogenesis, lympho-angiogenesis, and cancer stem cell functions (reviewed in [50]). FAK inhibition in a murine model resulted in decreased proliferation and migration of tumor-associated Mφ [51]. Notch signaling plays context-dependent roles in differentiation, proliferation, and apoptosis, and has been implicated in cancer progression through influencing both the FAK and NFκB activity [52,53]. In the context of Mφ, our results in this study show that along with an increase in Notch1 expression, FAK level was also increased in splenic Mφ of Chagas mice and in Mφ in vitro infected with *T. cruzi*, and treatment with SRT1720 inhibited the expression as well as phosphorylation of FAK (Figure 6). Treatment with SRT1720 or FAK inhibitor also suppressed the NFκB activity in Mφ infected by *Tc*, thus, suggesting that SIRT1 downregulation of proinflammatory Mφ profile in Chagas mice is through inhibition of FAK and NF-κB activity. Others have shown that deletion of SIRT1 increased the expression of FAK in Mφ [43] and resveratrol treatment suppressed the cell proliferation and activation of FAK [54]. We surmise that *Tc* inhibition of SIRT1 promotes the Notch-FAK-NF-κB signaling pathway and contributes to pathological proinflammatory activation of Mφ in chronic Chagas disease.

Our finding of no effects of SRT1720 on parasite persistence but a decline in proinflammatory activation of Mφ indicates that factors other than parasites provide stimulus for Mφ proinflammatory activation in chronic CD. Indeed, we have shown that extracellular vesicles released in sera of Chagas patients with clinically symptomatic heart disease (in comparison to seropositive individuals with no clinical symptoms of heart disease) elicited a proinflammatory profile in human Mφ [25]. In another study, we found that cardiac lysates of Chagas mice consisted oxidized proteins, and incubation of Mφ with cardiac lysates of CD (vs. normal) mice elicited inflammatory cytokines and ROS response [55]. We postulate that SRT1720 treatment decreased the generation of damage associated molecules (e.g., oxidized cardiac proteins) and thereby removed the stimuli that contribute to proinflammatory activation of Mφ.

In summary, we have shown that SIRT1 activity regulates the FAK-dependent activation of transcription factors involved in proliferation, migration, and proinflammatory activation of yolk sac Mφ in chronic CD, and SIRT1 agonists offer a potential therapeutic approach to reprogram the proinflammatory Mφ and control chronic inflammatory pathology in CD.

## 5. Conclusions

The pathological role of T cells in chronic Chagas disease is well studied. However, very few reports document the role of macrophages in Chagas cardiomyopathy. In this study, we demonstrate that immune cells of yolk-sac origin contribute to proinflammatory macrophage profile in Chagas disease. We also found that proinflammatory macrophages can be reprogrammed to healing phenotype through enhancing the SIRT1 activity that inhibits the FAK signaling of factors involved in macrophage proliferation and proinflammatory activation in Chagas disease.

## Supplementary Materials

The following are available online at https://www.mdpi.com/2073-4409/9/1/80/s1, Figure S1: Splenic characterization of HSC monocytes in Chagas mice, Table S1: List of antibodies used in this study, Table S2: Oligonucleotides used in this study.
