# Supplementary material for: Origin of Monocytes/Macrophages Contributing to Chronic Inflammation in Chagas Disease: SIRT1 Inhibition of FAK-NFκB-Dependent Proliferation and Proinflammatory Activation of Macrophages
Source: Cells. 2019 Dec 28;9(1):80. doi: 10.3390/cells9010080 (PMC7017093; doi:10.3390/cells9010080)
Supplement: Supplementary file 1 [file cells-09-00080-s001.pdf]

Supplemental Files

S1 Table: List of antibodies used in this study

Antigen	Fluorochrome	Clone	Cat #	Source	Figure #
**Flow cytometry**					
CD16/CD32	-	2.4G2	53142	BD Biosci	1 & 2
CD115 (MCSFR)	APC	AFS98	17-1152-82	eBiosci	1C
CD117 (cKit)	PE	2B8	553355	BD Biosci	1C & 1D
CD135	PE-CF594	A2F10.1	562537	BD Biosci	1D
Ly6C	Alexa Flour 488	HK1.4	128022	BioLegend	1E
Ly6G	eFluor 450	RB6-8C5	48-5931-82	eBiosci	1E
Fixable viability dye	eFluor	-	65-0865-14	eBiosci	2
CD11b/Mac1	APC/Cy7	M1/70	557657	BD Biosci	2B
CD80	APC	16-10A1	104714	Biolegend	3A & 3E
CD64	Alexa Fluor 647	X54-5/7.1	558539	BD Biosci	3B & 3F
CD206	PE	C068C2	141706	Biolegend	3C & 3G
CD200	PE	OX110	12-5201-82	eBiosci	3D & 3H
F4/80	PerCP/Cy5.5	BM8	123128	Biolegend	3E & 5M
**Western blotting and immunohistochemistry**					
CD11b	-	2LPM19c	sc-20050	Santa Cruz	3M & 5E
CD80 (B7-2)	-	D-6	sc-28347	Santa Cruz	3M & 5E
CD206	-	15-2	sc-58986	Santa Cruz	3M & 5E
F4/80	-	C-7	sc-377009	Santa Cruz	3M & 5E
GAPDH	-	14C10	3683	Cell Signal	3M, 5E & 6A
iNOS	-	N-20	sc-651	Santa Cruz	5G
Arg-1	-	H-52	sc-20150	Santa Cruz	5G
CD11b/Mac1	-	SPM281	Ab75693	Abcam	5G
SIRT1	-	D60E1	3931	Cell Signal	6A
c-myb	-	D-7	sc-74512	Santa Cruz	6A&6C
FAK	-	D-1	Sc-271126	Santa Cruz	6A&6C
Pu.1	-	C-3	sc-390405	Santa Cruz	6A&6C
Phospho-FAK	-	Tyr397	3283	Cell Signal	6A&6C
RunX1	-	DW71	sc-101146	Santa Cruz	6A&6C
NF-κB-p65	-	F-6	sc-8008	Santa Cruz	6D
Lamin A/C	-	H-110	sc20681	Santa Cruz	6D

S2 Table. Oligonucleotides used in this study

Gene Name	Protein Name	Genbank Accession #	Primer name	Sequence 5'-3'	Size (bp)
Arg1	Arginase 1	NM_007482.3	Arg1 F<br>Arg1 R	CAGAAGAATGGAAGAGTCAG<br>CAGATAGCAGGGAGTCACC	249
CD11b	Integrin alpha M	NM_008401.2	CD11b F<br>CD11b R	GCAGTCATCTTGAGGAACCGTGTC<br>GTTGGTATTGCCATCAGCGTCC	195
CD80	CD80 antigen	NM_001359898.1	CD80 F<br>CD80 R	GGCAAGGCAGCAATACCTTA<br>CTCTTTGTGCTGCTGATTCG	94
CD206	Mannose receptor C-	XM_021155588.1	CD206 F<br>CD206 R	CCTGAACAGCAACTTGACCA<br>GCAATGGCCATAGAAAGGAA	268
F4/80	Adhesion G protein-	NM_001355722.1	F4/80 F<br>F4/80 R	CTTTGGCTATGGGCTTCCAGTC<br>GCAAGGAGGACAGAGTTTATCGTG	165
GAPDH	Glyceraldehyde 3-	NC_000067.6	GAPDH F<br>GAPDH R	TGGCAAAGTGGAGATTGTTG<br>TTCAGCTCTGGGATGACCTT	402
IL-6	Interleukin-6	NM_001314054.1	IL-6F<br>IL-6R	TTCTCATTTCCACGATTTCCCAG<br>TTCCATCCAGTTGCCTTCTTG	175
IL-10	Interleukin-10	NM_010548.2	IL-10 F<br>IL-10R	GCTCTTACTGACTGGCATGAG<br>CGCAGCTAGGAGCATGTG	103
TNF-α	Tumor necrosis factor α	NC_000083.6	TNF-α F<br>TNF-α R	GTTCTATGGCCCAGACCCTCACA<br>TACCAGGGTTTGAGCTCAGC	836
Tc18Sr	*T. cruzi* 18S ribosomal	NC_018331.1	*Tc*18S F<br>*Tc*18S R	TTTT GGGC AACA GCAG GTCT<br>CTGC GCCT ACGA GACA TTCC	200

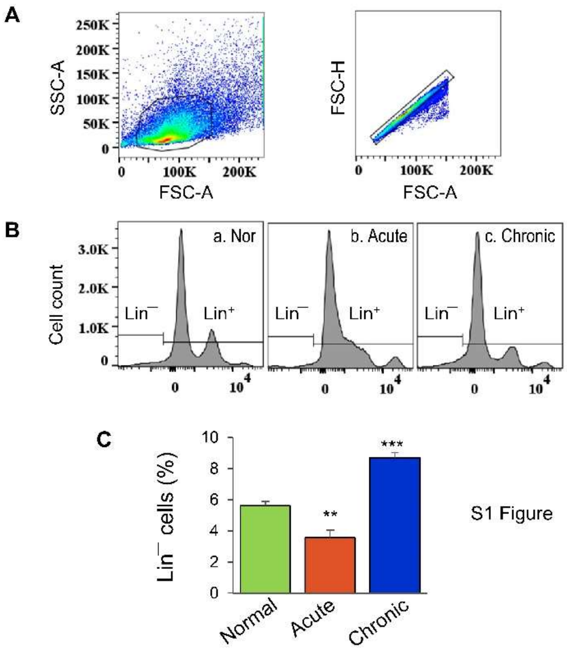

S1 Figure. Splenic characterization of HSC monocytes in Chagas mice. C57BL/6 mice were infected with *T. cruzi* (10,000 trypomastigotes per mouse, i.p.) and euthanized at 30 days and 150 days post-infection (pi) corresponding to acute infection phase and chronic heart disease phase, respectively. Single cell suspensions of splenic cells were stained with fluorescence-conjugated antibodies. Shown are representative flow cytometry images of spleen cells gated in forward and side scatter area (**A**, left panel) and forward scatter height to exclude doublets (**A**, right panel). Then cells were further gated for Lin$^-$ and Lin$^+$ phenotype **(B)**. Bar graph shows the mean percentages of Lin$^-$ splenocytes in normal, and acutely

infected and chronically infected mice. Data are representative of two independent experiments (n=3 mice per group per experiment, > 2 flow cytometric observations per splenic sample) and plotted as mean value ± SEM. Statistical significance (*Tc* infection vs. no infection) is annotated as $^{**}p \leq 0.01$ and $^{***}p \leq 0.001$.
